# Domoic Acid Transfer to Milk: Evaluation of a Potential Route of Neonatal Exposure

**DOI:** 10.1289/ehp.7649

**Published:** 2005-01-20

**Authors:** Jennifer M. Maucher, John S. Ramsdell

**Affiliations:** Marine Biotoxins Program, Center for Coastal Environmental Health and Biomolecular Research, National Oceanic and Atmospheric Administration–National Ocean Service, Charleston, South Carolina

**Keywords:** amnesic shellfish poisoning, domoic acid, ELISA, milk, plasma, urine

## Abstract

Domoic acid (DA), produced by the diatom genus *Pseudo-nitzschia*, is a glutamate analog and a neurotoxin in humans. During diatom blooms, DA can contaminate filter-feeding organisms, such as shellfish, and can be transferred by ingestion to higher trophic levels. Several intoxication events involving both humans and various marine mammals have been attributed to DA. Affected organisms show neurological symptoms such as seizures, ataxia, headweaving, and stereotypic scratching, as well as prolonged deficits in memory and learning. Neonatal animals have been shown to be substantially more sensitive to DA than adults. However, it has not been demonstrated whether DA can be transferred to nursing young from DA-exposed mothers. This study demonstrates transfer of DA from spiked milk (0.3 and 1.0 mg/kg) to the plasma of nursing neonatal rats and an overall longer DA retention in milk than in plasma after 8 hr in exposed dams. DA was detectable in milk up to 24 hr after exposure (1.0 mg/kg) of the mothers, although the amount of DA transferred to milk after exposure was not sufficient to cause acute symptoms in neonates.

The marine biotoxin domoic acid (DA), produced by the cosmopolitan diatom genus *Pseudo-nitzschia*, is responsible for causing amnesic shellfish poisoning (ASP). DA can affect many different species, most often by the contamination of shellfish beds and planktivorous fish via filter feeding. Subsequent consumption by other organisms can lead to accumulation of DA in higher trophic levels, including avian, marine mammal, and human populations (Gulland et al. 2002; Lefebvre et al. 1999; Scholin et al. 2000; Work et al. 1993). The consequent effects of DA exposure in these organisms are multiple, with previous research focusing on the behavioral and neurological effects of exposure.

Symptomatic responses to DA exposure have been documented in mice, rats, and cynomolgus monkeys (Iverson et al. 1989; Tasker et al. 1991; Tryphonas et al. 1990a, 1990b) with the most common behavioral symptoms being scratching, tremors, and seizures at varying doses of DA. These responses also parallel those observed from environmental exposures in sea lions (Gulland et al. 2002). Various researchers have also reported that DA exposure causes prolonged neuroexcitation and extensive degeneration in the hippocampus as well as more rostral areas of the brain, including the septum and olfactory bulb (Peng and Ramsdell 1996; Peng et al. 1994; Scallet et al. 1993, 2004). DA-induced damage to hippocampal formation has been correlated with both learning (Clayton et al. 1999) and memory deficits in humans and experimental animals (Nakajima and Potvin 1992; Petrie et al. 1992; Sutherland et al. 1990; Teitelbaum et al. 1990).

Most of these previous experiments have dealt with mature subjects, leaving the impact of DA exposure on fetal development relatively poorly understood and studied. Dakshinamurti et al. (1993) demonstrated that intrauterine exposure of DA indicated age-related developmental neurotoxicity in mice, with hippocampal necrosis observable after 30 days of age. Xi et al. (1997) reported that neonatal rats were substantially more susceptible to DA exposure than adults. Because these animals maintained higher blood levels of DA, Xi et al. (1997) proposed that susceptibility resulted from insufficient renal clearance of toxin. The susceptibility diminished as a function of neonatal age (Doucette et al. 2000; Xi et al. 1997), corresponding to the maturation of renal function.

The rapidity with which DA clears from an exposed organism’s blood (> 99% in 4 hr) (Suzuki and Hierlihy 1993; Truelove and Iverson 1994) is also a factor when assessing DA exposure. This fast clearance makes it difficult to detect DA after exposure within a suitable time frame, especially when considered from a biomonitoring stance. The prevalence of this marine biotoxin and its effects on marine mammal and human populations makes biomonitoring capability an important consideration, especially understanding the impact of maternal exposure and the subsequent effects on neonates as a susceptible population.

Because it is known that DA is neurotoxic and can affect neonatal more readily than adult rats (Xi et al. 1997), our study focused on whether DA could be transferred from DA-exposed rat mothers (dams) to the young through milk. The presence of DA was measured using an antibody-based approach (Garthwaite et al. 1998) via direct competitive enzyme-linked immunosorbant assay (cELISA) with a limit of detection < 0.01 ng/mL. The cELISA has been used successfully to detect low DA concentrations in blood extracts and plasma from laboratory exposures (Maucher and Ramsdell, in press). Additionally, we explored the partitioning of DA in milk, plasma, and urine of exposed dams, and the clearance of DA from each matrix.

## Materials and Methods

### Rat exposure: dams.

All animal exposures were performed by Argus Research (Horsham, PA), a division of Charles River Laboratories, Inc. Twelve female Crl:CD (SD)IGS BR VAF/Plus rats, 318–418 g, and their respective litters, were provided by Charles River Laboratories, Inc. (Horsham, PA), and dams were divided into three treatment groups. Groups 1 and 2 were injected intraperitoneally (ip) with a non-lethal dose of 1.0 mg/kg (0.1 mg/mL) DA (Sigma, St. Louis, MO) in phosphate-buffered saline (PBS) on day 12 of lactation. Group 3 control mice were injected with an equal volume of PBS. This dose was chosen because the threshold for neurological symptoms in adult rats is between 1.0 and 2.0 mg/kg (Tryphonas et al. 1990b). Blood was collected from the jugular vein 1, 4, 8, and 24 hr after exposure, transferred into dipotassium EDTA-coated tubes, and centrifuged. Plasma was collected and frozen (−68 to −78°C) until shipment for analysis. After blood collection, dams were given one unit oxytocin to stimulate lactation approximately 5 min before collection of milk samples. Milk was collected from group 1 dams at 1, 8, and 24 hr after dosing, from group 2 dams at 4 hr after dosing, and from group 3 control dams at 24 hr after dosing. Milk from the group 2 and group 3 collection points was put aside for either direct or DA-spiked feeding, respectively, to group 1 and group 2 pups; the remaining samples were then frozen until shipment for analysis. Urine samples were also collected from the dams throughout the following time intervals: 0–4 hr, 4–8 hr, and 8–24 hr after dosing from group 1 dams and 2–24 hr after dosing from group 3 (control) dams.

### Rat exposure: pups.

Postnatal day (PND) 12 pups from the dams described above were also divided into three groups. Milk from group 3 control dams was spiked at 0.3 and 1.0 mg/kg and given to pup groups 1 and 2, respectively, via oral gavage. Group 3 pups were treated via oral gavage with milk collected at hour 4 from group 2 dams (1.0 mg/mL exposed). Under isoflurine anesthesia, pup blood was collected 2 hr after dosing via cardiac puncture, centrifuged to collect plasma, and the plasma frozen (−68 to −78°C) until shipment for analysis. The 1.0 mg/kg dose was previously determined by preliminary range-finding experiments (1.0–8.0 mg/kg) to be at the lowest observable effect level (LOEL) for neonatal rats (PND10) using oral exposure. This LOEL was based on a modified Tasker scoring system (Tasker et al. 1991), with head tremors being the most common behavioral response of pups in the 1.0-mg/kg group, and a dose-dependent increase in severity of symptoms (scratching, whole body tremors, convulsions) with the higher dosed pups (data not shown).

### ASP direct cELISA kit.

We used ASP direct cELISA kits (Kleivdal H et al., unpublished data) from Biosense Laboratories (Bergen, Norway; www.biosense.com) to analyze all plasma, urine, and milk samples. This kit uses a polyclonal ovine anti-DA antibody (Garthwaite et al. 1998) conjugated to horseradish peroxidase (HRP), to which free DA in samples or standards compete with DA-conjugated proteins coated on the plate well surface. Samples were incubated with the antibodies, washed with PBS with 0.05% Tween 20 (PBST), and then treated with tetramethylbenzidine, which reacts with the HRP enzyme to form a blue end-product. Addition of 0.3 M H_2_SO_4_ turned the blue to yellow, and the plate was read on a FluoStar plate reader (BMG Labtechnologies, Durham, NC) at 450 nm. Analyses of DA concentrations in the standard curve and samples were done using ELISA data processing software provided by Biosense Laboratories. Additional analyses were done using Prism 4.0 (Graphpad, San Diego, CA).

### Sample dilutions.

Samples were diluted either 1:10 or 1:100 before being run on the cELISA. Plasma samples run at 1:10 were compared by *t*-test (Prism 4.0; Graphpad) to a matrix control at the same dilution, due to matrix effects at this low dilution, to qualitatively express the presence or absence of DA. Milk samples were quantified on a standard curve with milk matrix present at a 100-fold dilution for each standard concentration. We ran other standard curves in the presence of urine and milk at 100-fold dilutions to assess any matrix effects.

## Results

### Matrix effects.

**Figure 1:**
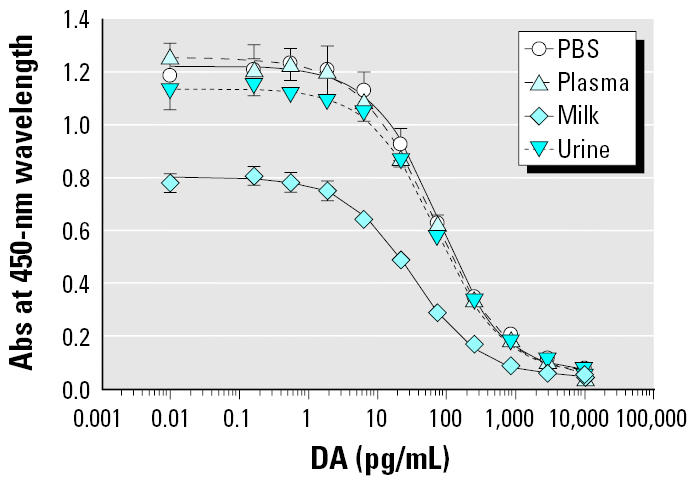
Comparison of DA standard curves run with PBS and in the presence of 100-fold dilutions of plasma, milk, and urine matrices. Abs, absorbance values. Error bars represent SDs (*n* = 2).

When the Biosense kit standard curve, run in PBST, was compared to the same standard curve run in the presence of plasma, urine, and milk matrices, each at 100-fold dilutions for each standard concentration, resulting EC_50_ (half-maximal effective) values were 75.70, 65.15, 72.85, and 33.34 pg/mL, respectively (Figure 1). A 33% decrease in the maximum absorbance value (A_max_) was observed between the standard curve and the 1:100 milk standard curve, indicating a matrix effect in milk, along with the lowered EC_50_. The other matrices at the same 1:100 dilution showed no such matrix effect. A similar decrease in A_max_ absorbance was also determined previously in blood diluted 10-fold (data not shown).

### Clearance of DA from blood.

**Figure 2:**
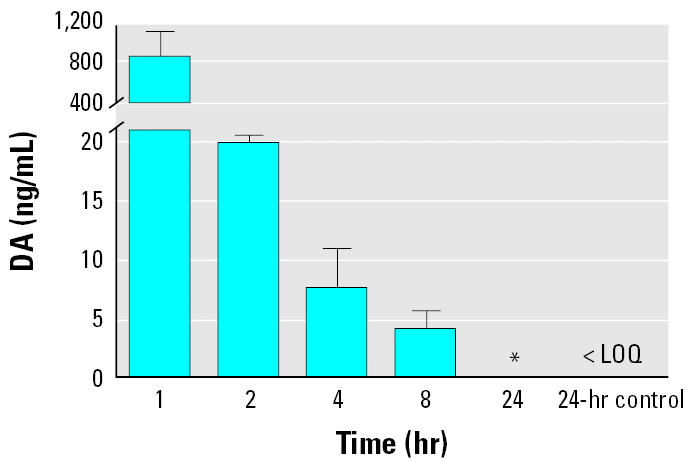
DA concentrations in blood of exposed dams (1.0 mg/kg) over time (*n* = 6). The limit of quantification (LOQ) for a sample was 1.0 ng/mL.
*Indicates detectable but nonquantifiable DA via a statistically significant difference in mean from the control as determined by paired *t*-test (*p* = 0.004).

The exposed dams showed a rapid clearance of DA from plasma, with > 99% eliminated in the first hour (Figure 2). However, 4 ng/mL was still quantifiable after 8 hr, and DA was still detectable at 24 hr (Figure 2) when compared to the control rat (group 3) plasma at the same 1:10 dilution as the treated rats (groups 1 and 2; *p* < 0.005). There was a significant difference between raw absorbances of the control plasma extract and the treated dams at 24 hr, with a 10-fold dilution each (*p* < 0.005; Figure 2). These results indicate the ability to detect the presence of DA in plasma 24 hr after exposure and to quantify the amount of DA present at ≤ 8 hr after exposure.

### Clearance from milk.

**Figure 3:**
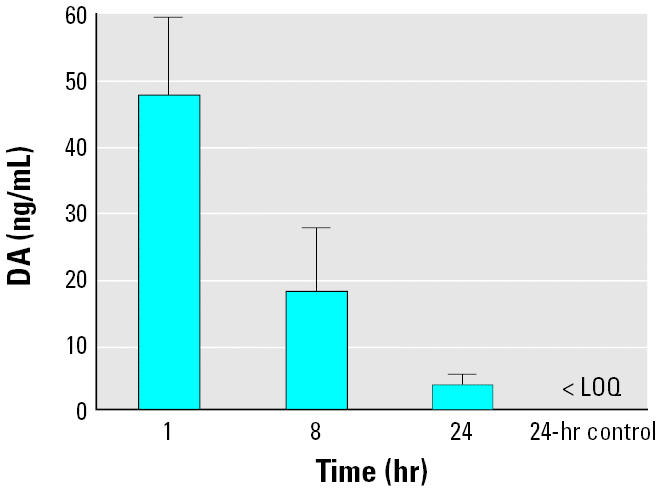
Concentrations of DA in milk of exposed dams (1.0 mg/kg) at three time points. Errors bars represent SDs (*n* = 4), and each time point was significantly different from the control (one-way analysis of variance with Dunnett’s post-test; *p* < 0.05). The limit of quantification (LOQ) for a sample was 1.0 ng/mL.

**Figure 4:**
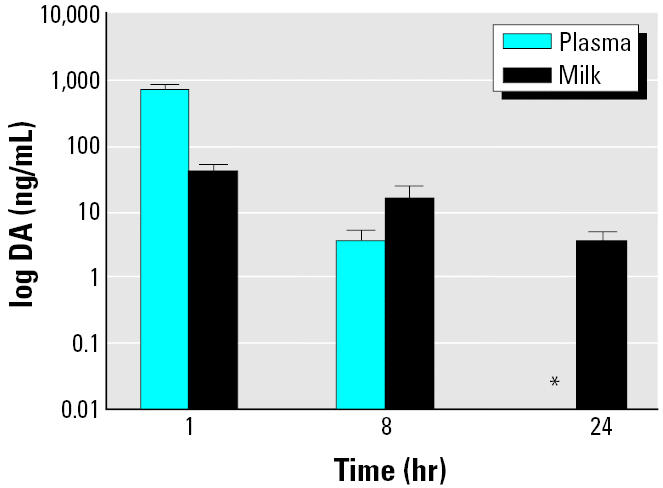
Comparison of DA concentrations over time in milk and plasma of exposed dams (1.0 mg/kg). Concentrations are in log scale.
*Indicates detectable but nonquantifiable DA via a statistically significant difference in mean from the control as determined by paired *t*-test (*p* = 0.004).

There was a matrix effect evident in the 100-fold diluted milk samples, so we analyzed the milk samples using a standard curve in the presence of milk at the same 100-fold dilution to accurately ascertain the DA concentrations (data not shown). The amount of DA present in milk decreased rapidly, with < 1% still detectable within the first 2 hr; however, 3 ng/mL DA were still measurable at 24 hr (Figure 3). There was only a 20% change in DA concentration between the 1- and 8-hr time points, in contrast to the > 99% change in the concentration of DA in the plasma extracts between the same time points (Figure 4). There was 4-fold greater DA retained in milk than plasma at the 8-hr time point, and 3 ng/mL DA still quantifiable at 24 hr, whereas DA was only detectable in plasma at 24 hr (Figure 4). This indicates a longer retention time of DA in milk than in plasma.

### Clearance from urine.

**Figure 5:**
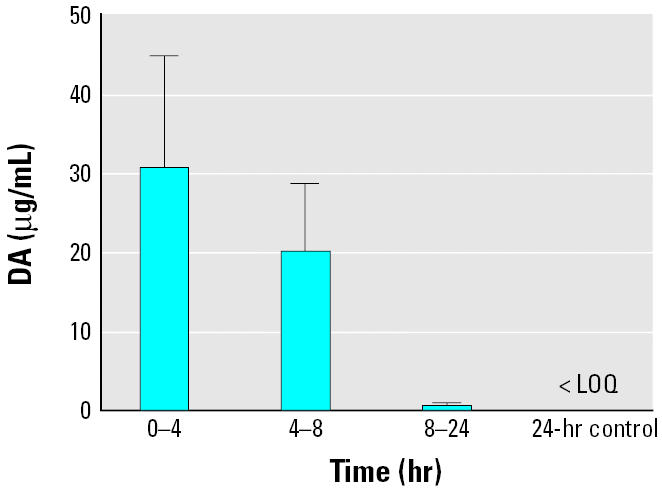
DA concentrations in urine of exposed dams (1.0 mg/kg) over time. Error bars represent SDs (*n* = 4), and each time point was significantly different from the control (one-way analysis of variance with Dunnett’s post-test; *p* < 0.001). The limit of quantification (LOQ) for a sample was 1.0 ng/mL.

The DA concentrations found in urine within the first hour after dosing were 3 orders of magnitude higher than any of the other matrices. Initial DA values were 310 μg/mL during the 0–4 hour time period, 210 μg/mL during the 4–8 hr collection period, and decreased to approximately 6 μg/mL between 8 and 24 hr (Figure 5). There was no detectable DA in the 2–24 hr control.

### Transfer from dam to pup.

**Figure 6:**
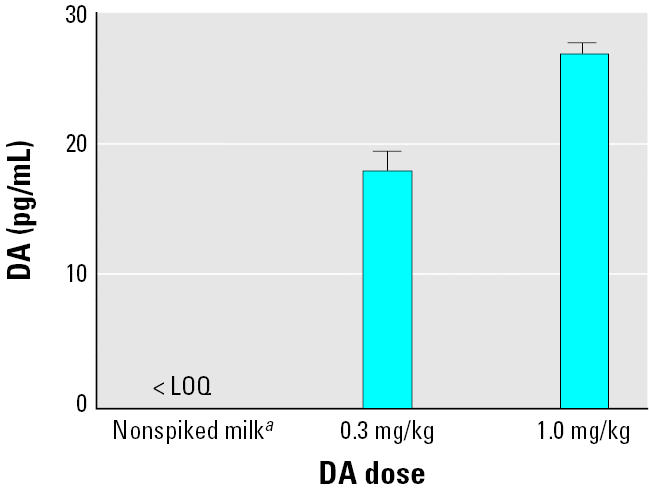
DA concentrations in blood of postnatal day 12 rat pups exposed to either spiked milk or milk from exposed dams. Error bars represent SDs (*n* = 6).
***^a^***Milk from group 2 dams 4 hr after exposure (1.0 mg/kg) given directly to pups without spiking. The limit of quantification (LOQ) for a milk sample was 0.3 ng/mL.

There was no detectable DA in the plasma of pups that had been given milk collected from group 2 dams 4 hr after exposure to 1.0 mg/kg, whereas pups exposed to the DA-spiked milk (0.03 and 1.0 mg/kg) did have measurable amounts of DA in plasma (Figure 6). Two hours after exposure, > 99% of DA had cleared from plasma of pups from both the 0.03-mg/kg and the 1.0-mg/kg groups (Figure 6).

## Discussion

DA mortality events have become prevalent in recent years, especially in marine mammals, including sea lions, sea otters, and dolphins, off the coast of California. Although it is known that DA is transferred to higher trophic levels by seafood consumption, it is not known whether DA can also be transferred from an intoxicated mother to her young through milk. The purpose of this research was to determine how DA is partitioned in various body fluids after exposure to a sublethal dose and whether transfer of DA is possible through milk.

Our results showed that after a 1.0-mg/kg DA intraperitoneal exposure to lactating rats, there was a large range in retention of DA with time among the three body fluids tested. Results of DA partitioning among the three experimental body fluids of the exposed dams (plasma, urine, and milk) showed that most of the DA is excreted through the urine, which had initial 1-hr values 3 orders of magnitude greater than that in plasma, which, in turn had 10 times higher DA than that found in milk. This supports previous assertions that most systemic DA is cleared quickly through the kidneys. Suzuki and Hierlihy (1993) determined a clearance rate through rat kidneys of approximately 9.12 mL/min per kg body weight, and a half-life of roughly 20 min was reported by Truelove and Iverson (1994). Evidence for renal clearance also came from older patients that ingested DA-contaminated mussels in Canada in 1987 (Perl et al. 1990), as those with compromised kidney function were more severely affected by the DA exposure.

With the kidneys filtering much of the DA from the blood, less DA was detectable in the plasma of the exposed dams. More than 99% was cleared within 4 hr, which is similar to previous results by Peng and Ramsdell (1996) and Maucher and Ramsdell (in press). Hence, the clearance of DA from plasma is rapid, which would ordinarily make biomonitoring the plasma of exposed organisms difficult were it not for the sensitive detection method used. The cELISA enabled us to measure plasma concentrations of DA 8 hr after exposure, based on the EC_20_ value of the Biosense software used for analysis, and also to detect but not quantify DA present at 24 hr by comparison to a control.

The results of DA retention in milk were interesting for two reasons. First, although the DA concentrations in milk were 16 times lower than that in plasma at the 1-hr time point, by 8 hr (i.e., milk collected between the 1-hr and 8-hr interval) there was a four-fold higher quantity remaining in the milk compared to the plasma. Second, using the cELISA, there was still a quantifiable concentration of DA in milk collected in the 8–24 hr interval, whereas at the same time point in plasma, DA was detectable but not measurable. Because DA is retained longer in milk than in plasma, milk should be a useful fluid for biomonitoring DA exposure in lactating animals and humans. These results also initially suggested to us that, even in light of the rapid clearance, DA could potentially be transferred to feeding neonates and thus put them at higher risk for abnormal development, although our additional data failed to support this hypothesis.

Neonates exposed to DA-spiked milk absorbed the toxin, and the DA was measurable in their plasma. The amount of toxin spiked into the milk (1.0 mg/kg) was determined by preliminary range-finding experiments to be at the LOEL for neonatal rats using oral exposure (data not shown). This dose, and the lower dose of 0.3 mg/kg, led to measurable levels of plasma DA in the neonates at 2 hr. However, in this experiment, there was no detectable DA in the plasma of the neonates administered milk collected from dams exposed to 1.0 mg/kg DA 4 hr earlier. The amount of DA that accumulated in the milk over this first 4-hr collection period (60 ng/mL) would yield an oral dose of 240 ng/kg for a single 0.1-mL oral administration. Therefore, this oral dosage yields plasma levels in the neonate well below the limit of detection for DA.

We initially wanted to know if lactation was a relevant route of exposure for DA toxicity to neonates for several reasons. DA has potent developmental and neurological effects during early life stages. Neonatal rats have been shown to be up to 40 times more susceptible to DA exposure than adults (Xi et al. 1997), most likely due to underdeveloped renal filtration of the toxin. Incomplete development of the blood–brain barrier in neonates may also render them more susceptible to the effects of DA than adults (Preston and Hynie 1991). Doucette et al. (2003) showed that even low doses of DA (20 μg/kg) between PND8 and PND14 caused neurobehavioral changes such as early eye opening and decreased activity levels. Wang et al. (1999) showed that subcutaneous DA doses of 0.33 mg/kg, similar to our lowest dose by gavage, given to PND7 rats were enough to induce hindlimb paralysis, tremors, and death. A decrease of DA toxicity with increasing age of neonatal rats has also been determined by Xi et al. (1997) and Doucette et al. (2000), meaning that the youngest are the most susceptible to neuronal damage and death.

Clearly, neonates are much more susceptible to systemic administration of DA; however, DA administered orally to adult rats and mice is approximately 10-fold less potent (Iverson et al. 1990). This likely results because, as an acidic molecule, DA administered orally enters the bloodstream by rapid absorption through the gastric mucosa but to a lesser extent though the intestines. Indeed, Iverson et al. (1990) reported that most orally administered DA is eliminated in the feces. Comparison of the effects of DA administered orally in milk also indicates that it is about 10-fold less potent than systemic administration to neonatal rats (Maucher and Ramsdell, unpublished data). Our results indicate that the amount of DA a neonate may be exposed to following a single feeding (60 ng/kg) from a dam receiving 1.0 mg/kg is about 1/4,000th of an oral dose causing observable symptoms in neonates.

The findings presented here demonstrate that DA was transferable from the plasma to milk in lactating rats and was retained for longer periods of time in the milk compared to plasma. DA-spiked milk was absorbed by neonates and reached measurable levels in plasma, although the amount of DA transferred to the pups from the milk of exposed dams was well below symptomatic levels. These results indicate that this experimental acute DA exposure event in neonatal rats, although too low to be detectable in milk from exposed dams, was also insufficient to cause any acute observable effects in neonates. Additionally, these results provide a method to quantify DA in milk, which should be applicable to human public health concerns regarding breastfeeding infants, as well as wildlife exposure assessment.

## References

[b1-ehp0113-000461] Clayton EC, Peng YG, Means LW, Ramsdell JS (1999). Working memory deficits induced by single but not repeated exposures to domoic acid. Toxicon.

[b2-ehp0113-000461] Dakshinamurti K, Sharma SK, Sundaram M, Watanabe T (1993). Hippocampal changes in developing postnatal mice following intrauterine exposure to domoic acid. J Neurosci.

[b3-ehp0113-000461] Doucette TA, Bernard PB, Yuill PC, Tasker RA, Ryan CL (2003). Low doses of non-NMDA glutamate receptor agonists alter neurobehavioural development in the rat. Neurotoxicol Teratol.

[b4-ehp0113-000461] Doucette TA, Strain SM, Allan GV, Ryan CL, Tasker RAR (2000). Comparative behavioral toxicity of domoic acid and kainic acid in neonatal rats. Neurotoxicol Teratol.

[b5-ehp0113-000461] Garthwaite I, Ross KM, Miles CO, Hansen RP, Foster D, Wilkins AL (1998). Polyclonal antibodies to domoic acid, and their use in immunoassays for domoic acid in sea water and shell-fish. Nat Toxins.

[b6-ehp0113-000461] Gulland F, Haulena M, Fauquier D, Langlois G, Lander ME, Zabka T (2002). Domoic acid toxicity in California sea lions (*Zalophus californianus*): clinical signs, treatment, and survival. Vet Rec.

[b7-ehp0113-000461] Iverson F, Truelove J, Nera E, Tryphonas L, Campbell J, Lok E (1989). Domoic acid poisoning and mussel-associated intoxication: preliminary investigations into the response of mice and rats to toxic mussel extract. Food Chem Toxicol.

[b8-ehp0113-000461] Iverson F, Truelove J, Nera E, Tryphonas L, Campbell J, Lok E (1990). The toxicology of domoic acid administered systematically to rodents and primates. Can Dis Wkly Rep.

[b9-ehp0113-000461] Lefebvre KA, Powell CL, Busman M, Doucette GJ, Moeller PDR, Silver JB (1999). Detection of domoic acid in northern anchovies and California sea lions associated with an unusual mortality event. Nat Toxins.

[b10-ehp0113-000461] Maucher JM Ramsdell JS In press. Ultrasensitive detection of domoic acid in mouse blood by competitive ELISA using blood collection cards. Toxicon. 10.1016/j.toxicon.2005.01.002 15777957

[b11-ehp0113-000461] Nakajima S, Potvin JL (1992). Neural and behavioral effects of domoic acid, an amnesic shellfish toxin, in the rat. Can J Psychol.

[b12-ehp0113-000461] Peng YG, Ramsdell JS (1996). Brain *fos* induction is a sensitive biomarker for the lowest observed neuroexcitatory effects of domoic acid. Fundam Appl Toxicol.

[b13-ehp0113-000461] Peng YG, Taylor TB, Finch RE, Switzer RC, Ramsdell JS (1994). Neuroexcitatory and neurotoxic actions of the amnesic shellfish poison, domoic acid. Neuro Report.

[b14-ehp0113-000461] Perl TM, Bedard L, Kosatsky T, Hockin JC, Todd ECD, Remis RS (1990). An outbreak of toxic encephalopathy caused by eating mussels contaminated with domoic acid. N Engl J Med.

[b15-ehp0113-000461] Petrie BF, Pinsky C, Standish NM, Bose R, Glavin GB (1992). Parenteral domoic acid impairs spatial learning in mice. Pharmacol Biochem Behav.

[b16-ehp0113-000461] Preston E, Hynie I (1991). Transfer constants for blood-brain barrier permeation of the neuroexcitatory shellfish toxin, domoic acid. Can J Physiol Pharmacol Neurol Sci.

[b17-ehp0113-000461] Scallet AC, Binienda ZK, Caputo FA, Hall S, Paule MG, Rountree RL (1993). Domoic acid-treated cynomolgus monkeys (*M. fascicularis*): effects of dose on hippocampal neuronal and terminal degeneration. Brain Res.

[b18-ehp0113-000461] Scallet AC, Kowalke PK, Rountree RL, Thorn BT, Binienda ZK (2004). Electrocephalographic, behavioral, and c-*fos* responses to acute domoic acid exposure. Neurotoxicol Teratol.

[b19-ehp0113-000461] Scholin CA, Gulland F, Doucette GJ, Benson S, Busman M, Chavez FP (2000). Mortality of sea lions along the central California coast linked to a toxic diatom bloom. Nature.

[b20-ehp0113-000461] Sutherland RJ, Hoesing JM, Whishaw IQ (1990). Domoic acid, an environmental toxin, produces hippocampal damage and severe memory impairment. Neurosci Lett.

[b21-ehp0113-000461] Suzuki CAM, Hierlihy SL (1993). Renal clearance of domoic acid in the rat. Food Chem Toxicol.

[b22-ehp0113-000461] Tasker RAR, Connell BJ, Strain SM (1991). Pharmacology of systemically administered domoic acid in mice. Can J Physiol Pharmacol.

[b23-ehp0113-000461] Teitelbaum JS, Zatorre RJ, Carpenter S, Gendron D, Evans AC, Gjedde A (1990). Neurologic sequelae of domoic acid intoxication due to the ingestion of contaminated mussels. N Engl J Med.

[b24-ehp0113-000461] Truelove J, Iverson F (1994). Serum domoic acid clearance and clinical observations in the cynomolgus monkey and Sprague-Dawley rat following a single IV dose. Bull Environ Contam Toxicol.

[b25-ehp0113-000461] Tryphonas L, Truelove J, Iverson F (1990a). Acute parenteral neurotoxicity of domoic acid in cynomolgus monkeys (*M. fascicularis*). Toxicol Pathol.

[b26-ehp0113-000461] Tryphonas L, Truelove J, Nera E, Iverson F (1990b). Acute neurotoxicity of domoic acid in the rat. Toxicol Pathol.

[b27-ehp0113-000461] Wang GJ, Schmued LC, Andrews AM, Scallet AC, Slikker W, Binienda ZK (1999). Systemic adminstration of domoic acid-induced spinal cord lesions in neonatal rats. J Spinal Cord Med.

[b28-ehp0113-000461] Work TM Beale AM Fritz L Quilliam MA Silver M Buck K 1993. Domoic acid intoxication of brown pelicans and cormorants in Santa Cruz, California. In: Toxic Phytoplankton Blooms in the Sea (Smayda TJ, Shimizu Y, eds). Amsterdam:Elsevier, 643–650.

[b29-ehp0113-000461] Xi D, Peng YG, Ramsdell JS (1997). Domoic acid is a potent neurotoxin to neonatal rats. Nat Toxins.

